# Supplementary material for: Airway Spatial Transcriptomics in Smoking
Source: medRxiv. 2025 Apr 3:2025.04.01.25325047. Preprint. [Version 1] doi: 10.1101/2025.04.01.25325047 (PMC11998807; doi:10.1101/2025.04.01.25325047)
Supplement: Supplement 1 [file media-1.pdf]

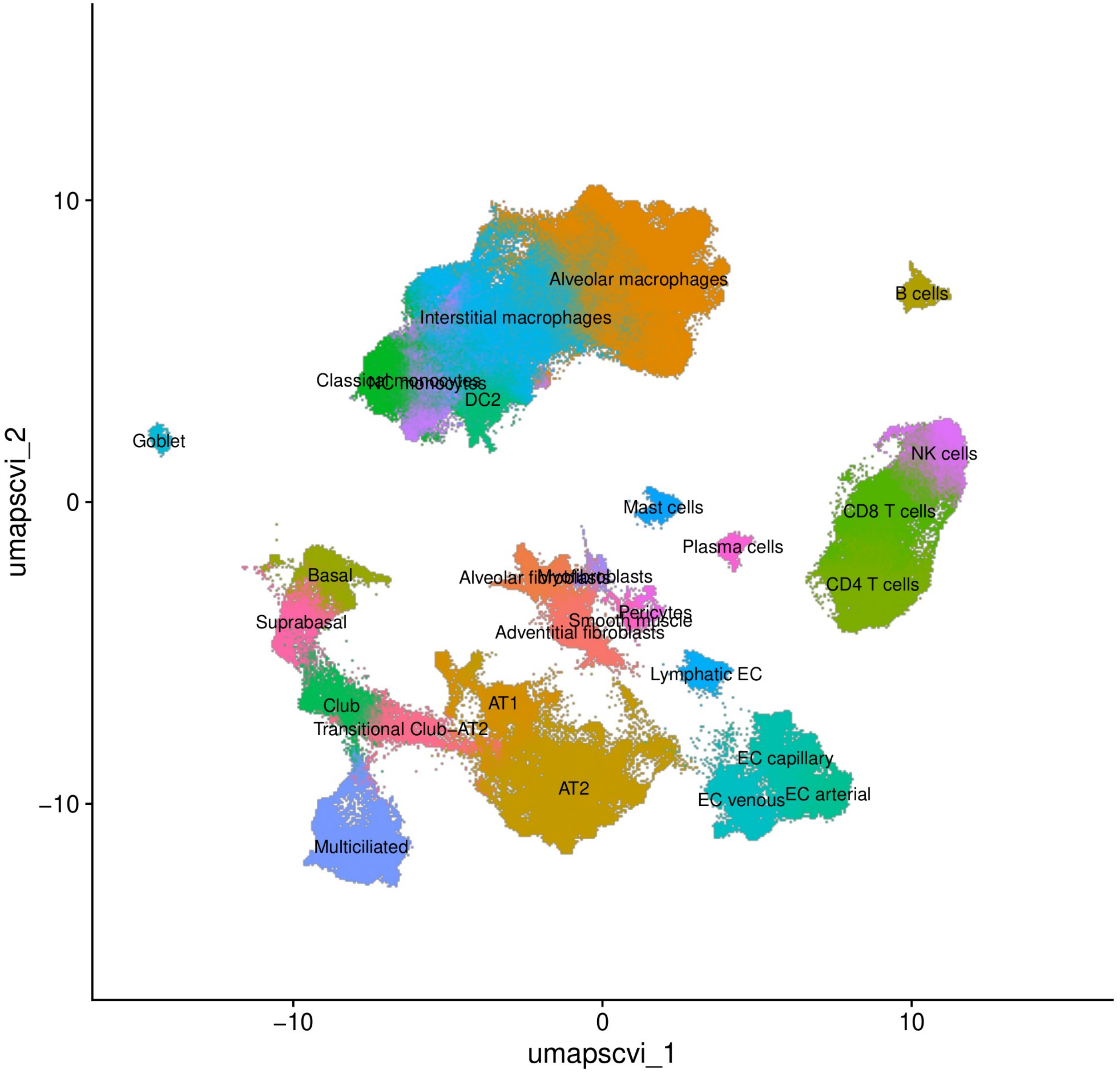

Figure S1. UMAP of scRNA-seq atlas

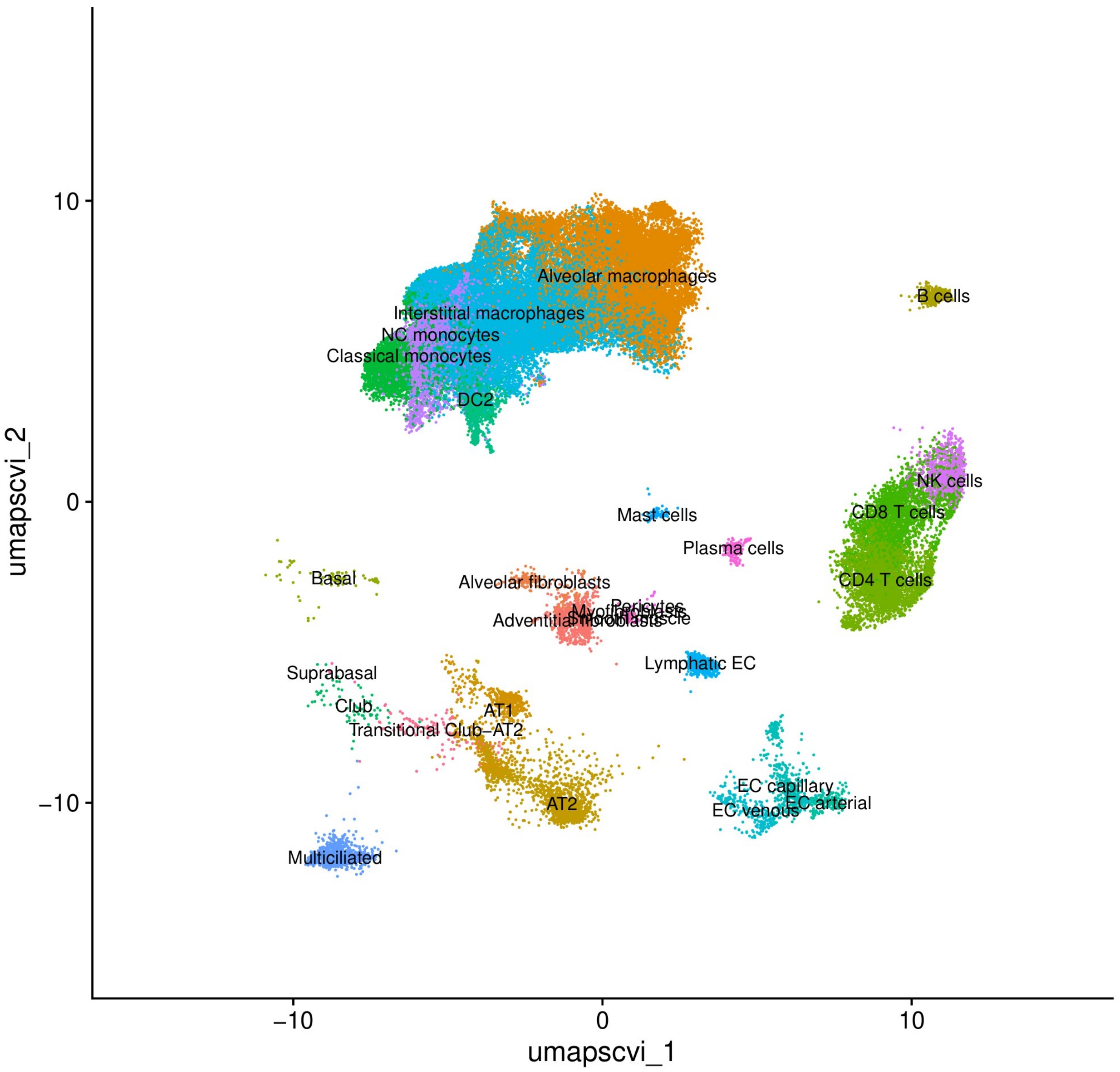

Figure S2. UMAP of Sauler et al. scRNA-seq data in atlas

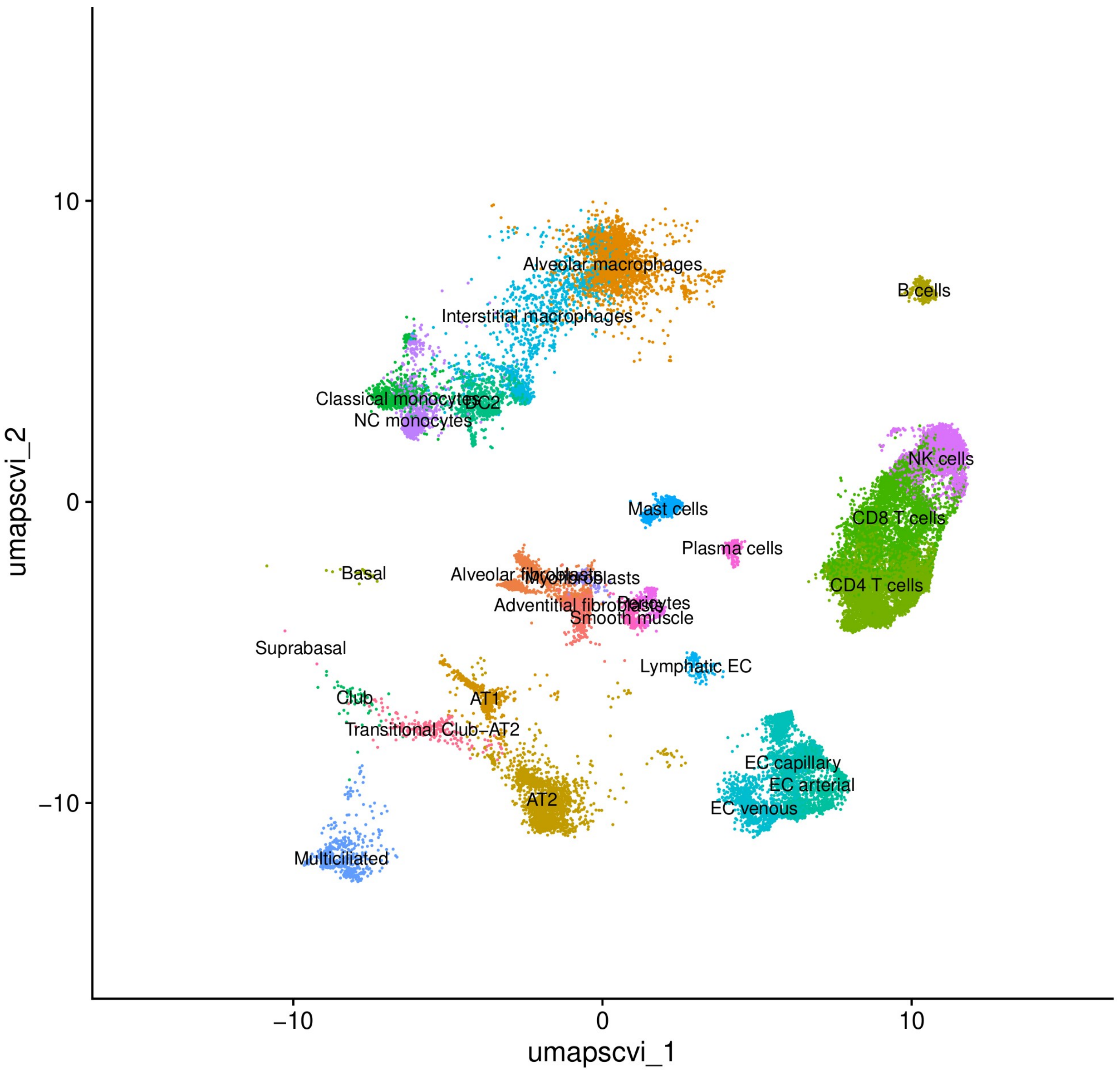

Figure S3. UMAP of Watanabe et al. scRNA-seq data in atlas

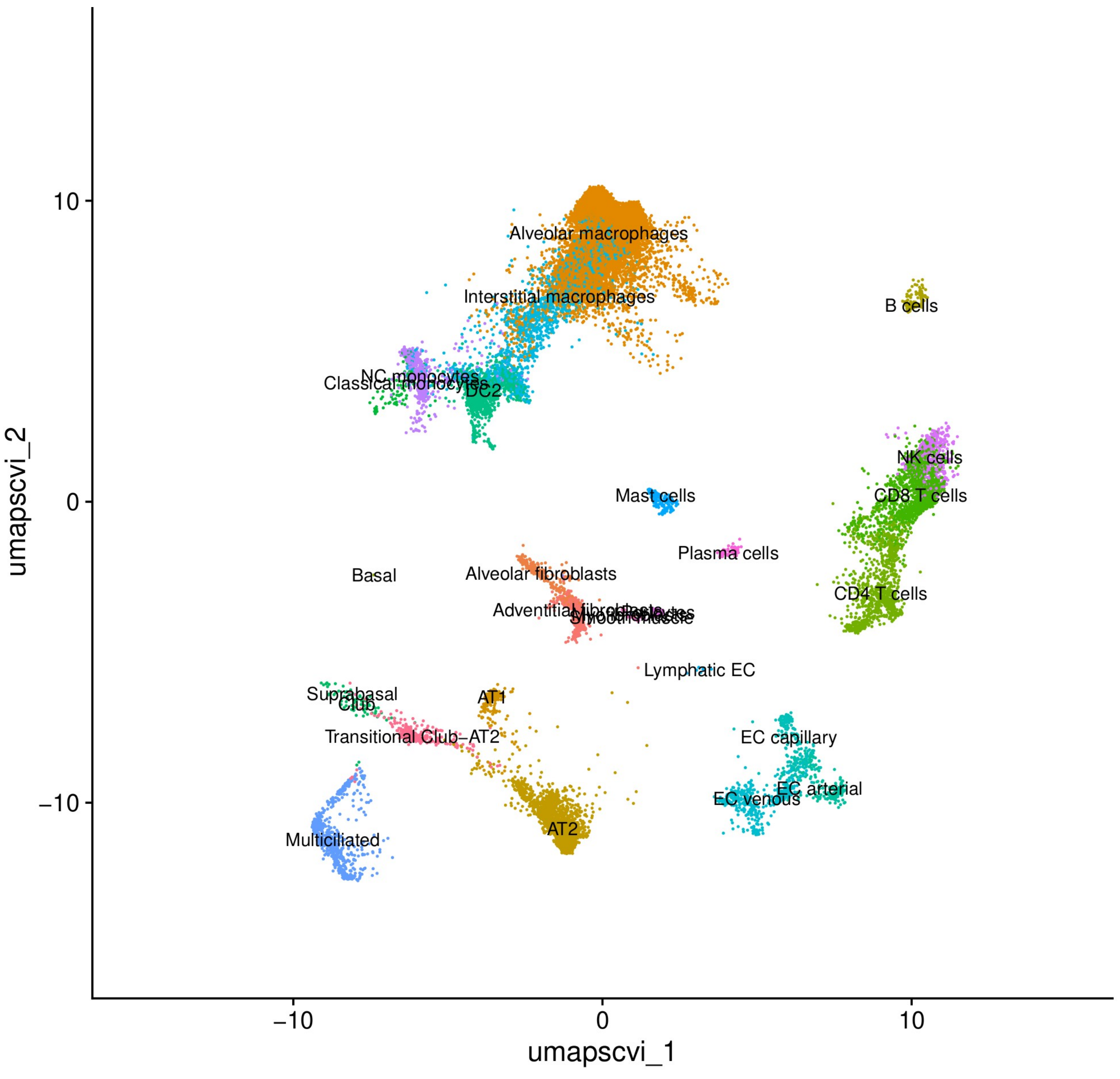

Figure S4. UMAP of Huang et al. scRNA-seq data in atlas

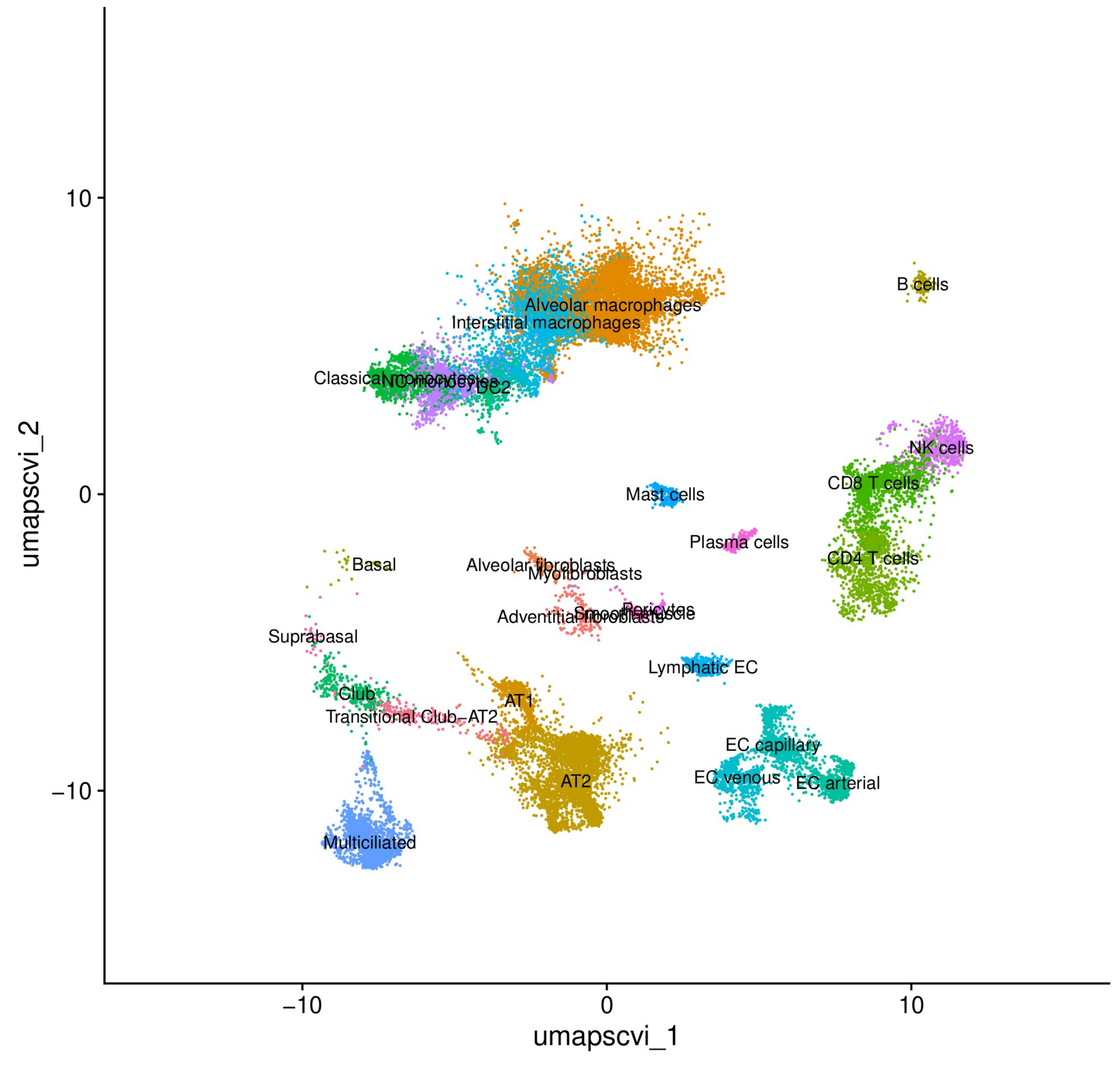

Figure S5. UMAP of Habermann et al. scRNA-seq data in atlas

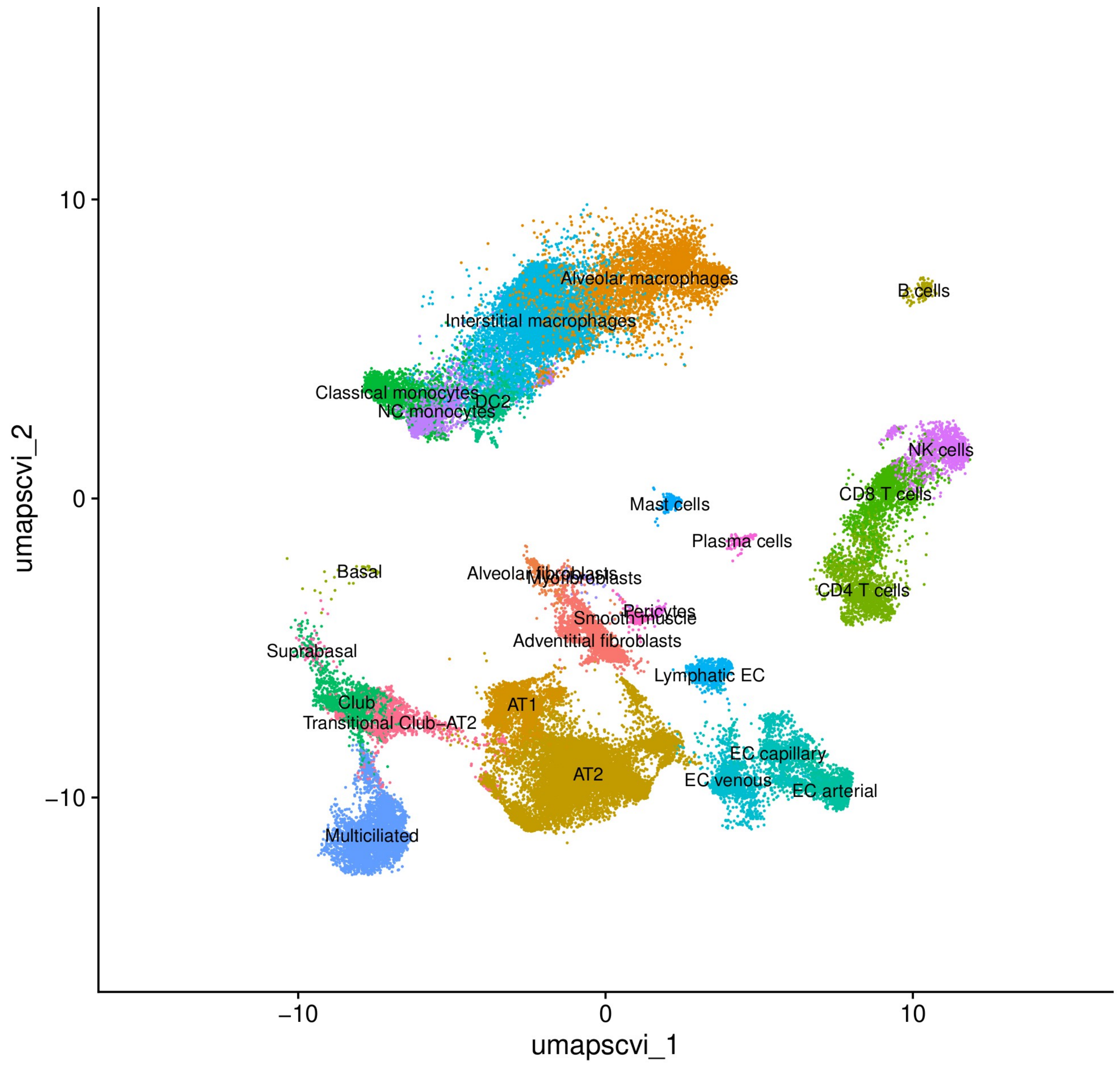

Figure S6. UMAP of Natri et al. scRNA-seq data in atlas

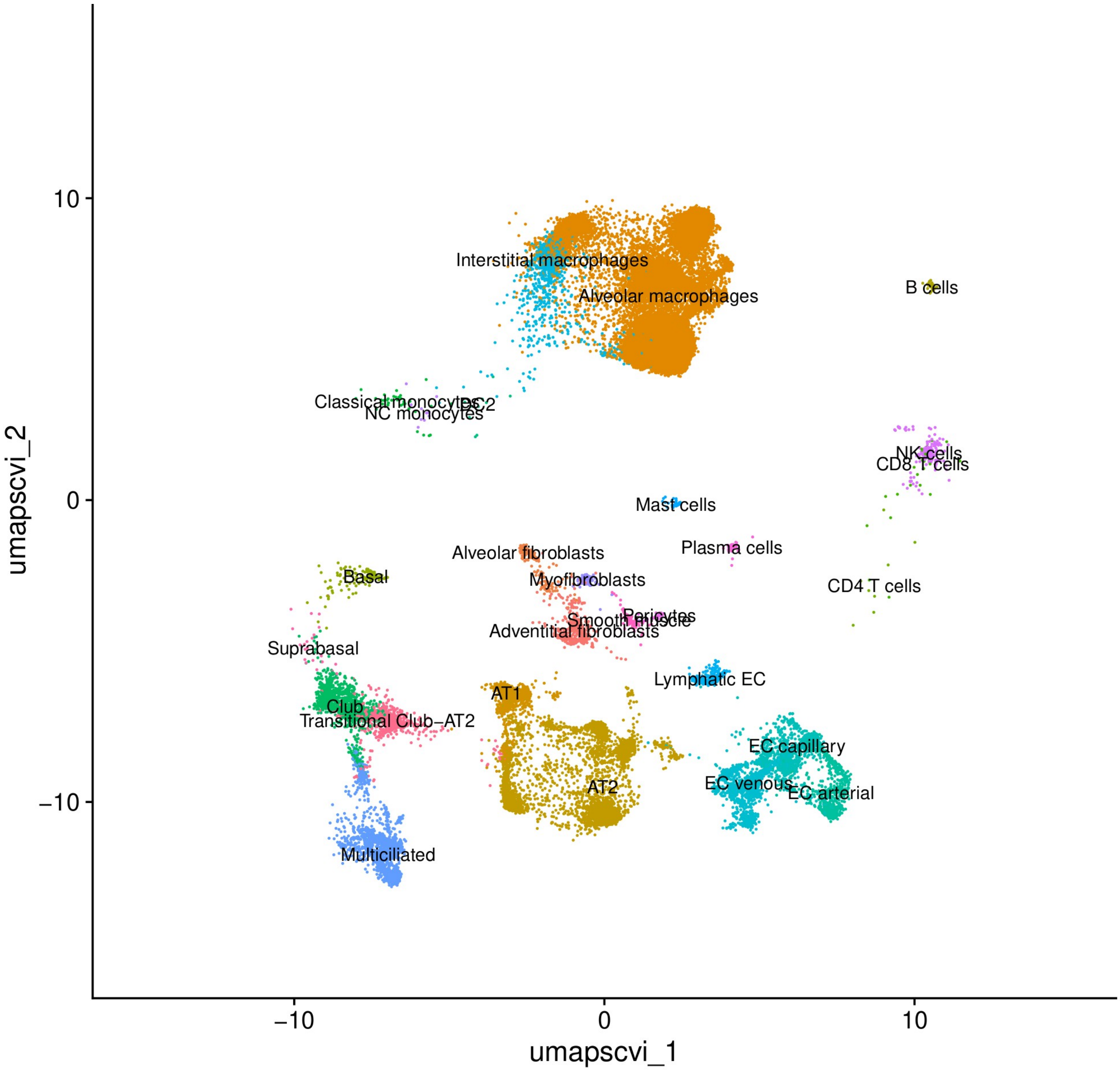

Figure S7. UMAP of Basil et al. scRNA-seq data in atlas

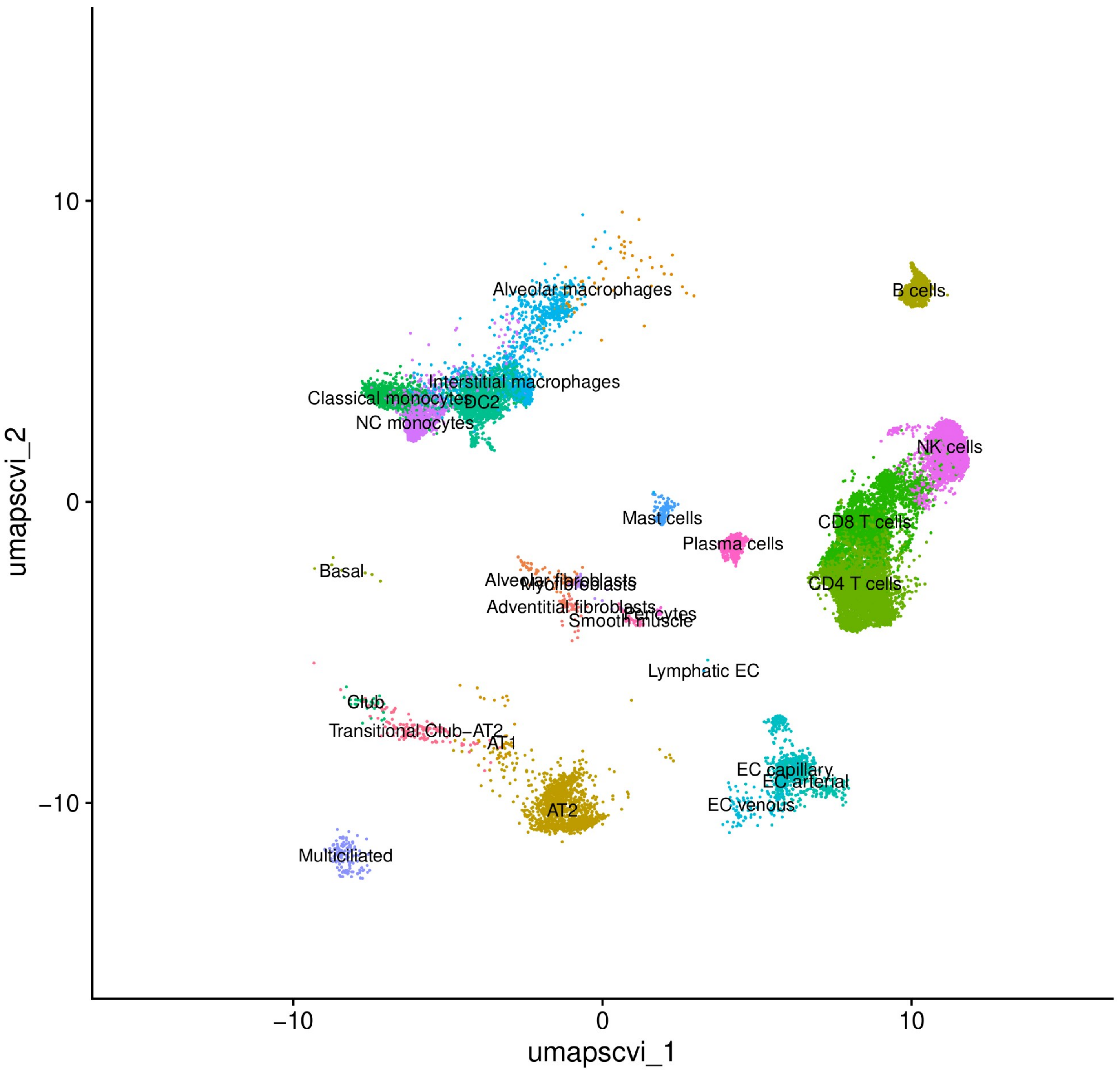

Figure S8. UMAP of Villaseñor-Altamirano et al. scRNA-seq data in atlas

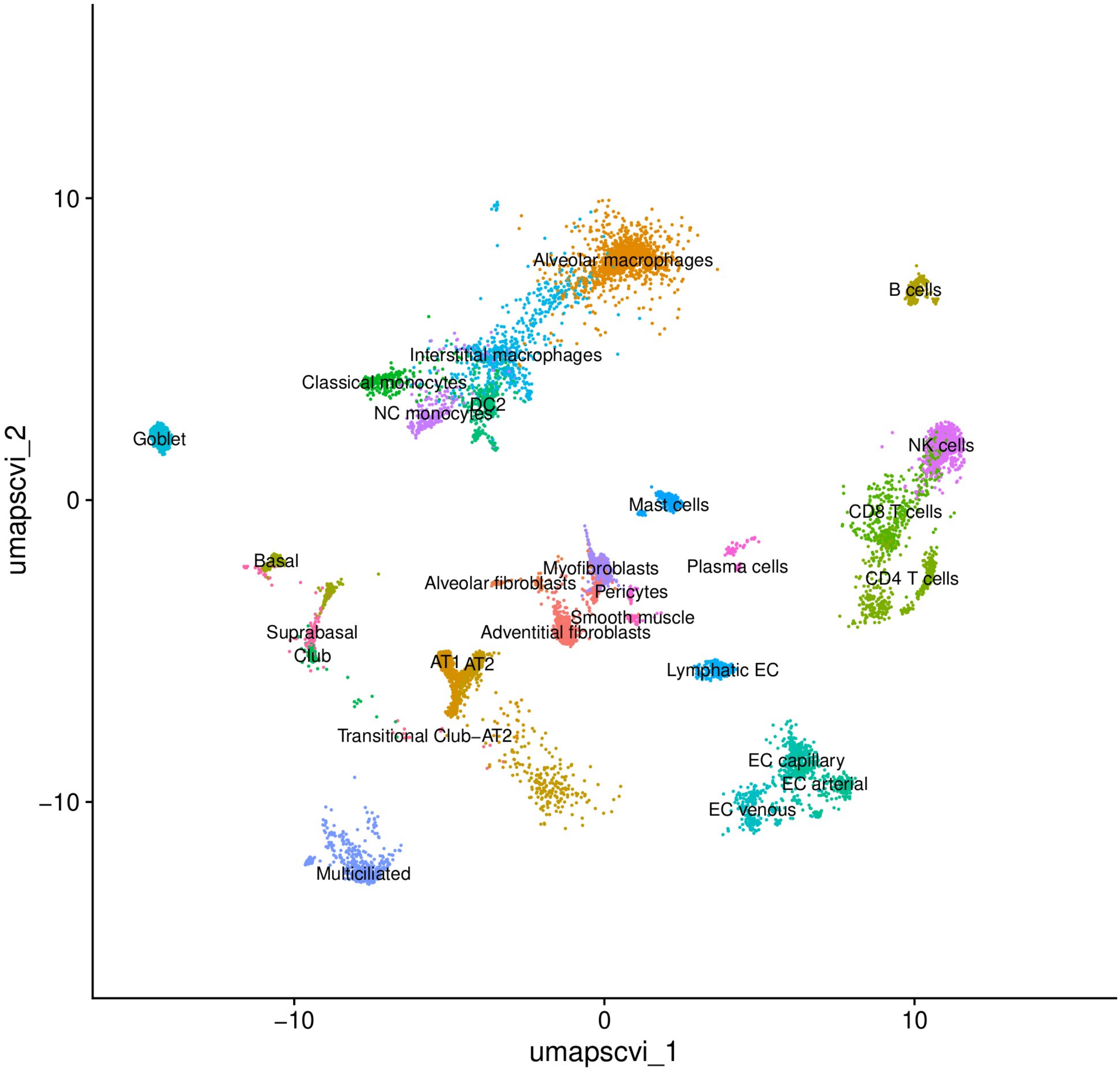

Figure S9. UMAP of Madissoon et al. scRNA-seq data in atlas

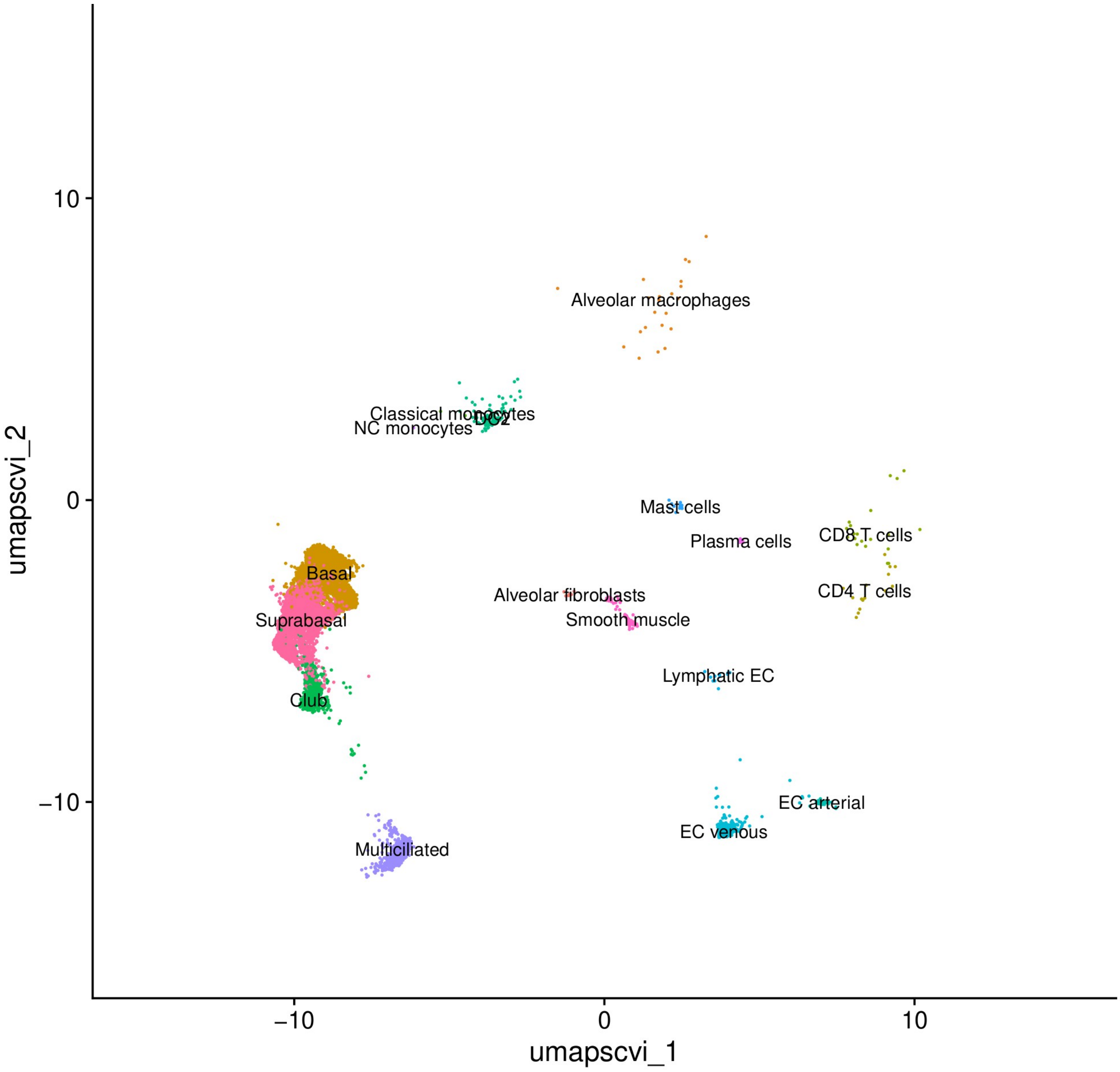

Figure S10. UMAP of Deprez et al. scRNA-seq data in atlas

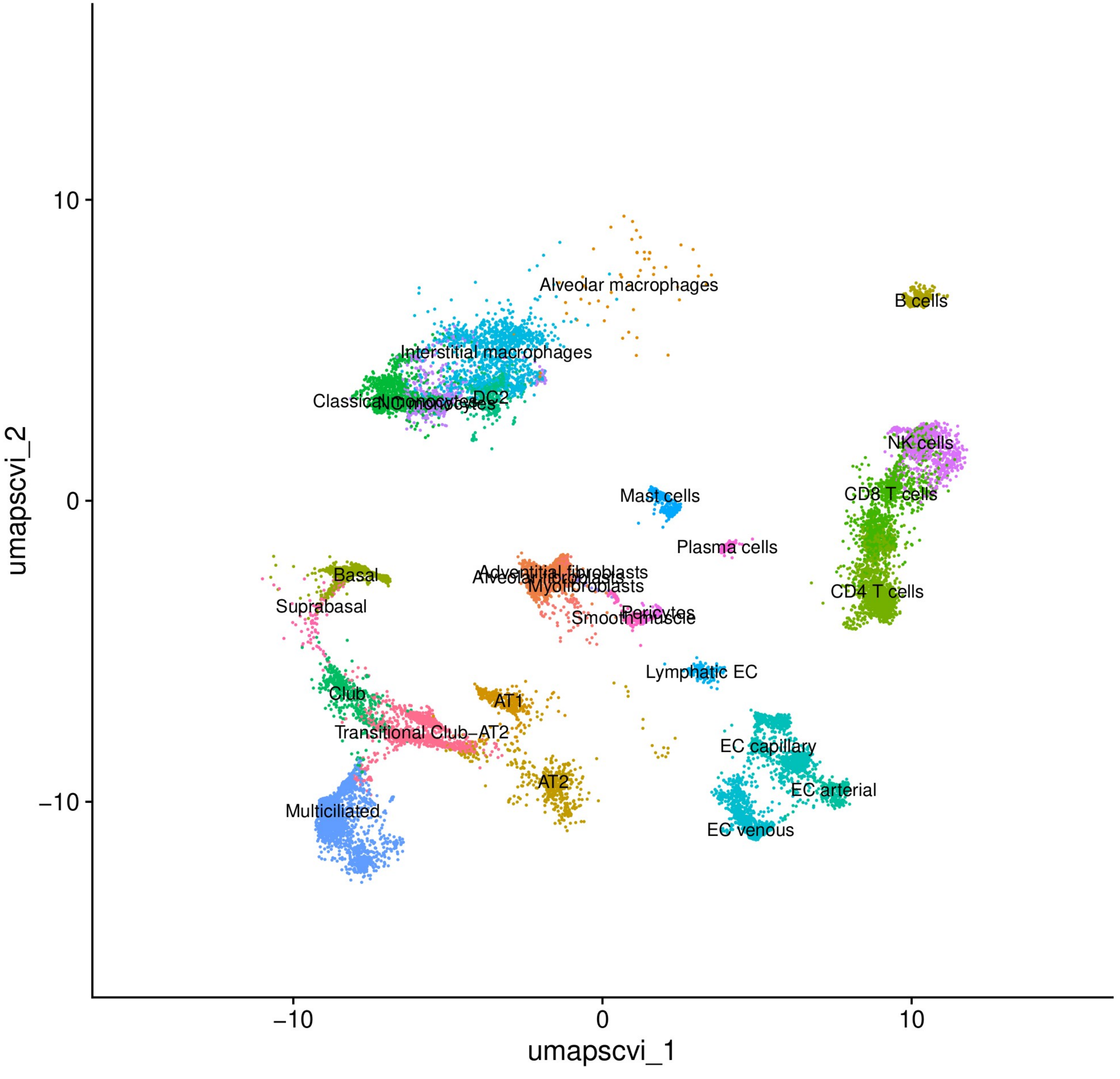

Figure S11. UMAP of Murthy et al. scRNA-seq data in atlas
