# Supplementary material for: Airway Spatial Transcriptomics in Smoking
Source: medRxiv. 2025 Apr 3:2025.04.01.25325047. Preprint. [Version 1] doi: 10.1101/2025.04.01.25325047 (PMC11998807; doi:10.1101/2025.04.01.25325047)
Supplement: Supplement 2 [file media-2.pdf]

Airway Spatial Transcriptomics in Smoking

Jarrett D. Morrow[1,2,#], Zaid W. El-Husseini[1,2], Jeong H. Yun[1,2] and Craig P. Hersh[1,2]

1. Channing Division of Network Medicine, Brigham and Women's Hospital, Boston, MA

2. Harvard Medical School, Boston, MA

# Corresponding Author:

Jarrett D. Morrow, Ph.D., M.S.

Channing Division of Network Medicine

Brigham and Women's Hospital

181 Longwood Avenue, Boston, MA 02115

jarrett.morrow@channing.harvard.edu

Supplemental Methods

Lung single-cell RNA-seq data

The lung scRNA-seq reference atlas for use with CytoSPACE was created by harmonizing data from ten prior lung tissue studies (n=104; Supplemental Table S1) [1–11]. These scRNA-seq and phenotypic data for each of these studies are publicly available from the Gene Expression Omnibus (GEO), Short Read Archive (SRA), and the published articles [1–11]. Madissoon and colleagues produced single-nucleus RNA-seq data for two never-smoker and two current-smoker subjects as part of their spatial transcriptomic study of lung tissue where they identified 80 lung cell types and states, and gleaned details regarding lung micro-environments [11]. In the study of healthy human airways, Deprez and colleagues created a single-cell atlas while investigating cell population distributions and transcriptional changes along the airways [10]. Murthy et al. investigated distal airways using spatial and single-cell transcriptomics and identified unique airway cell types and trajectories with lineage and developmental relevance, while creating a distal lung map [9]. In an extensive study of cell-type specific transcriptomics and cell types in COPD, Sauler and colleagues identified novel subtypes of alveolar epithelial type II (AT2) cells relevant to COPD, among many other findings [2,8]. Watanabe and colleagues also characterized lung AT2 cells using scRNA-seq [3], and Huang et al. observed a pro-inflammatory effect of monocytes on alveolar epithelial cells in a lung study of COPD [1]. Using scRNA-seq, Habermann and colleagues examined the role of epithelial and mesenchymal cell types in pulmonary fibrosis [6], and Natri and colleagues studied the genetic control of lung gene expression in pulmonary fibrosis [7]. Basil and colleagues identified a unique secretory cell type distinct from cells found in larger proximal airways using scRNA-seq analyses [5]. A multi-omic single-cell study by Villaseñor-Altamirano et al., where particular CD8+ T-cell states in the lung were implicated in mild-moderate COPD [4].

Single-cell RNA-seq data were available from GEO for six of the subjects in the Watanabe et al. [3] dataset (Supplemental Table S1). Sequencing data for two additional subject were obtained from the SRA repository. We mapped these sequencing data to the GRCh38 human genome reference using Cell Ranger (10x Genomics) to create the scRNA-seq gene expression count matrix for all cells from these subjects. These data were concatenated with the GEO expression data to create a single dataset for the Watanabe et al. study.

Within each of the study datasets, prior to harmonization, we removed cells with greater than 15% mitochondrial expression content or less than 1,000 transcripts. Cells identified as multiplets using Scrublet [12] were also excluded. Cell-type classifications for all remaining cells were obtained using the reference-based mapping pipeline Azimuth [13]. The Azimuth pipeline [13] matched expression profiles of each cell in the scRNA-seq data with specific cell types annotated in the scRNA-seq data from the Human Lung Cell Atlas [14]. After concatenating the annotated data from the ten studies (merging of ten Seurat objects in R), cell types with a mean cell count per subject less than ten were removed. Endothelial Cell (EC) general capillary and aerocyte capillary cells were grouped as EC capillary cells and EC venous pulmonary and EC venous systemic cells were grouped as EC venous cells. Lymphatic EC mature cells were relabeled as Lymphatic EC cells and Basal resting cells were relabeled as Basal. We exported the merged data in adata format and used the scVI method within the Python

single-cell variational inference tools (scvi-tools) package [15,16] to perform dimensionality reduction of the merged expression data. We created a Uniform Manifold Approximation and Projection (UMAP) using the scVI output to identify outlying cells not within cell-type clusters. A cluster was defined by an ellipse centered on the UMAP values for a particular cell type. The ellipse size was 2*MAD (median absolute deviation) along each axis of the ellipse. Cells outside each of their respective cell-type cluster ellipse were excluded from later analyses. A final UMAP plot was used to observe harmonization of the datasets.

Spatial transcriptomics

In this study, we used the publicly available Visium (10X Genomics) spatial transcriptomic data from the lung tissue study by Madissoon and colleagues [11]. A Seurat object was created using the spatial data for each sample using the R package Seurat [13]. Spots within the Visium data with fewer than 500 counts in the gene expression data were excluded from the analyses. Although data were available from lung parenchyma samples, this low-expression filtering step yielded too few spots for analysis in the data for these sample. Spatial transcriptomic data from three never-smoker bronchi samples and three current-smoker bronchi samples were available for analyses following this filtering (Supplemental Table S2). Within each sample, genes with zero counts across all spots were excluded from the dataset.

Spatial transcriptomic data at single-cell resolution

Integrating Spatial and Single-cell Transcriptomic data leverages the strength of both technologies to provide a spatially resolved perspective on tissue cell-cell communications at single-cell resolution. We used the Python package CytoSPACE to construct spatial datasets with high gene coverage and spatially-resolved scRNA-seq data [17], by mapping cells from the lung scRNA-seq reference atlas to spots in lung Visium transcriptomic data. For the current-smoker (CS) Visum samples, we used the cells within the scRNA-seq atlas from the ever-smoker subjects. Likewise, for the never-smoker (NS) samples, we used scRNA-seq data from the never-smokers. We downsampled the scRNA-eq data to create an atlas containing no more than 1,000 cells for each cell type. Only genes represented in both the scRNA-seq atlas and the spatial sample were included in the data used in the CytoSPACE mapping process. The cells mapped to each spot in the spatial transcriptomic output data are located at the center of their assigned spot. The mapped cell type proportions across the Visium spots for each sample were viewed using the plotSpatialScatterpie function in the R package SPOTlight [18], where use of color by cell type was made consistent across all samples. We examined the usage of cells during the mapping process with respect to study source using bar plots for both CS and NS samples, and tested for enrichment of particular study data using hypergeometric tests, after adjusting for the downsampling of atlas cells prior to CytoSPACE mapping. During quality assessment, we examined the mapped cell count distribution across all spots for both the NS and CS samples.

Cell-cell interactions

CellChat (v2) enables prediction of differential cell-cell communication by smoking using data from multiple spatial transcriptomics datasets [19]. The method was developed for use with spatial transcriptomics in a format similar to the Visium (10x Genomics) platform, with RNA-seq data stored for each spot within a grid that covers

the assayed tissue area. We have leveraged the capabilities of the CellChat method for use with the virtual single-cell transcriptomic data we created with CytoSPACE. Instead of a single set of RNA-seq data in each spot, our approach has scRNA-seq data for multiple mapped cells within each spot, with the mapped cells located at the center of their assigned spot. Likewise, the group label for each spot (typically created using a clustering method) was replaced by the cell types for each of the mapped cells from our lung scRNA-seq atlas. In this usage model, we are inferring spatially proximal cell-cell communication between interacting cell types from spatially resolved transcriptomics at virtual single-cell resolution.

In the analyses we used the CellChat human database, specifically the subset for only Cell-Cell Contact, ECM-Receptor, and Secreted Signaling communication. The three samples for each subject (CS and NS; Supplemental Table S2) were combined into one CellChat object to create two analytical output datasets for observation of smoking effects (CS vs. NS) on cell-cell communication. We inferred the cell-cell communication network in each subject using the computeCommunProb function in CellChat [19] with the triMean method for computing the average gene expression per cell group, an interaction range of 250 micrometers, and no distance constraints to compute communication probability. We then inferred the probability at the signaling pathway level using the CellChat function computeCommunProbPathway. Heatmaps were created using the netVisual_heatmap function to illustrate the differential number of interactions and interaction strength in the cell-cell communication network between the two subjects (NS vs. CS). Also with a focus on cell types, we created scatter plots of differential signaling using the CellChat function netAnalysis_diff_signalingRole_scatter, where the x-axis and y-axis represent the differential outgoing and incoming communication probability for each cell type and positive or negative values correspond to an increase or decrease in CS, respectively. To examine pathway relevance, we used the CellChat function netAnalysis_signalingRole_heatmap to create a heatmap of the relative signaling strength of the top signaling pathways across all cell types.

Seeking additional rigor and context for the impact of smoking on cell-cell communication, we identified signaling genes over-expressed by smoking condition for each cell type using the CellChat function identifyOverExpressedGenes, with a fold change threshold of 1.5 and p-value cutoff of 0.5/27 (27 being the number of cell types tested in the analysis). The netMappingDEG function mapped the differential expressed genes onto the cell-cell communication results, where only differential expression of the ligands (senders) was considered in the analyses. After filtering for up- and down-regulated ligands meeting the threshold cutoffs for p-value and fold change, the pathways of interest were the ones most represented among the final cell-cell communication findings. We created chord diagrams using the CellChat function netVisual_chord_gene to visually examine the differential cell-cell communication (cell types and genes) in the signaling pathways of interest. This function was modified to produce consistent color assignment for each cell type across chord diagrams for all pathways.

References

1. Huang Q, Wang Y, Zhang L, Qian W, Shen S, Wang J, et al. Single-cell transcriptomics highlights immunological dysregulations of monocytes in the pathobiology of COPD. Respir Res. 2022;23:367.

2. Sauler M, McDonough JE, Adams TS, Kothapalli N, Barnthaler T, Werder RB, et al. Characterization of the COPD alveolar niche using single-cell RNA sequencing. Nat Commun. 2022;13:494.

3. Watanabe N, Fujita Y, Nakayama J, Mori Y, Kadota T, Hayashi Y, et al. Anomalous Epithelial Variations and Ectopic Inflammatory Response in Chronic Obstructive Pulmonary Disease. Am J Respir Cell Mol Biol. 2022;67:708–19.

4. Villaseñor-Altamirano AB, Jain D, Jeong Y, Menon JA, Kamiya M, Haider H, et al. Activation of CD8+ T Cells in Chronic Obstructive Pulmonary Disease Lung. Am J Respir Crit Care Med. 2023;208:1177–95.

5. Basil MC, Cardenas-Diaz FL, Kathiriya JJ, Morley MP, Carl J, Brumwell AN, et al. Human distal airways contain a multipotent secretory cell that can regenerate alveoli. Nature. 2022;604:120–6.

6. Habermann AC, Gutierrez AJ, Bui LT, Yahn SL, Winters NI, Calvi CL, et al. Single-cell RNA sequencing reveals profibrotic roles of distinct epithelial and mesenchymal lineages in pulmonary fibrosis. Sci Adv. 2020;6:eaba1972.

7. Natri HM, Del Azodi CB, Peter L, Taylor CJ, Chugh S, Kendle R, et al. Cell-type-specific and disease-associated expression quantitative trait loci in the human lung. Nat Genet. 2024;56:595–604.

8. Adams TS, Schupp JC, Poli S, Ayaub EA, Neumark N, Ahangari F, et al. Single-cell RNA-seq reveals ectopic and aberrant lung-resident cell populations in idiopathic pulmonary fibrosis. Sci Adv. 2020;6:eaba1983.

9. Kadur Lakshminarasimha Murthy P, Sontake V, Tata A, Kobayashi Y, Macadlo L, Okuda K, et al. Human distal lung maps and lineage hierarchies reveal a bipotent progenitor. Nature. 2022;604:111–9.

10. Deprez M, Zaragosi L-E, Truchi M, Becavin C, Ruiz García S, Arguel M-J, et al. A Single-Cell Atlas of the Human Healthy Airways. Am J Respir Crit Care Med. 2020;202:1636–45.

11. Madissoon E, Oliver AJ, Kleshchevnikov V, Wilbrey-Clark A, Polanski K, Richoz N, et al. A spatially resolved atlas of the human lung characterizes a gland-associated immune niche. Nat Genet. 2023;55:66–77.

12. Wolock SL, Lopez R, Klein AM. Scrublet: Computational Identification of Cell Doublets in Single-Cell Transcriptomic Data. Cell Syst. 2019;8:281-291.e9.

13. Hao Y, Hao S, Andersen-Nissen E, Mauck WM, Zheng S, Butler A, et al. Integrated analysis of multimodal single-cell data. Cell. 2021;184:3573-3587.e29.

14. Sikkema L, Ramírez-Suástegui C, Strobl DC, Gillett TE, Zappia L, Madissoon E, et al. An integrated cell atlas of the lung in health and disease. Nat Med. 2023;29:1563–77.

15. Gayoso A, Lopez R, Xing G, Boyeau P, Valiollah Pour Amiri V, Hong J, et al. A Python library for probabilistic analysis of single-cell omics data. Nat Biotechnol. 2022;40:163–6.

16. Virshup I, Bredikhin D, Heumos L, Palla G, Sturm G, Gayoso A, et al. The scverse project provides a computational ecosystem for single-cell omics data analysis. Nat Biotechnol. 2023;41:604–6.

17. Vahid MR, Brown EL, Steen CB, Zhang W, Jeon HS, Kang M, et al. High-resolution alignment of single-cell and spatial transcriptomes with CytoSPACE. Nat Biotechnol. 2023;41:1543–8.

18. Elosua-Bayes M, Nieto P, Mereu E, Gut I, Heyn H. SPOTlight: seeded NMF regression to deconvolute spatial transcriptomics spots with single-cell transcriptomes. Nucleic Acids Res. 2021;49:e50.

19. Jin S, Plikus MV, Nie Q. CellChat for systematic analysis of cell–cell communication from single-cell transcriptomics. Nat Protoc. 2025;20:180–219.

Supplemental Tables

Table S1. Single-cell RNA-seq data sources with subject demographics

≈ 350,000 cells	Smoking Status (n=104)			
**Data Source ***	**Never-smokers**	**Ever-smokers**	**Female / Male**	**Repository**
Madissoon	2	2	2 / 2	https://5locationslung.cellgeni.sanger.ac.uk/
Deprez	10	0	6 / 4	GEO: GSE143868
Murthy	1	2	0 / 3	GEO: GSE178360
Sauler	22	6	12 / 16	GEO: GSE136831
Watanabe	3	5	3 / 5	GEO: GSE173896 (6 samples)<br>SRA: SRP318548 (2 samples)
Huang	4	2	0 / 6	GEO: GSE171541
Habermann #	2	8	3 / 7	GEO: GSE135893
Natri #	12	12	7 / 17	GEO: GSE227136
Basil	3	2	3 / 2	GEO: GSE168191
Villaseñor-Altamirano	4	2	5 / 1	https://zenodo.org/records/8393742
**Total Subjects**	**63**	**41**	**41 / 63**	

* First author of primary study article
# Control subjects only

Table S2. Summary of spatial transcriptomic samples with subject demographics

Subject ID	Smoking Status	Sex	Age (years)	Sample name*	Location
A42	Never-smoker	Male	60-64	WSA_LngSP8759311	Bronchi at the fourth generation
				WSA_LngSP8759312	
				WSA_LngSP8759313	
A37	Current-smoker	Female	55-60	WSA_LngSP9258468	Bronchi at the second/third generation
				WSA_LngSP10193348	
				WSA_LngSP9258464	

* EMBL-EBI BioStudies/ArrayExpress: E-MTAB-11640

Table S3. Cell type distribution in the lung scRNA-seq atlas

**Cell type**	**Ever smoker cell counts**	**Never smoker cell counts**	**Cell counts for all subjects**
Alveolar macrophages	27637	51321	78958
Interstitial macrophages	20744	27923	48667
AT2	17305	18820	36125
CD4 T cells	10314	11483	21797
CD8 T cells	8312	10702	19014
Multiciliated	6586	9627	16213
Classical monocytes	5933	7310	13243
NK cells	5314	7489	12803
NC monocytes	4116	7980	12096
EC capillary	5472	4419	9891
AT1	5215	3715	8930
Basal	1024	7448	8472
EC venous	3799	4222	8021
DC2	2853	4447	7300
EC arterial	3021	3007	6028
Club	1992	3815	5807
Adventitial fibroblasts	2045	3518	5563
Transitional Club-AT2	1707	3335	5042
Suprabasal	273	4586	4859
Alveolar fibroblasts	2586	1531	4117
B cells	1112	2757	3869
Lymphatic EC	1823	1706	3529
Mast cells	1822	978	2800
Plasma cells	637	1184	1821
Goblet	0	1088	1088
Smooth muscle	653	423	1076
Myofibroblasts	197	798	995
Pericytes	625	360	985
**Total Cells**	**143117**	**205992**	**349109**

Supplemental Figures

Figures S1-S11. UMAP plots of scRNA-seq atlas for all studies, and Sauler et al., Watanabe et al., Huang et al., Habermann et al., Natri et al., Basil et al., Villaseñor-Altamirano et al., Madissoon et al., Deprez et al., Murthy et al. subsets

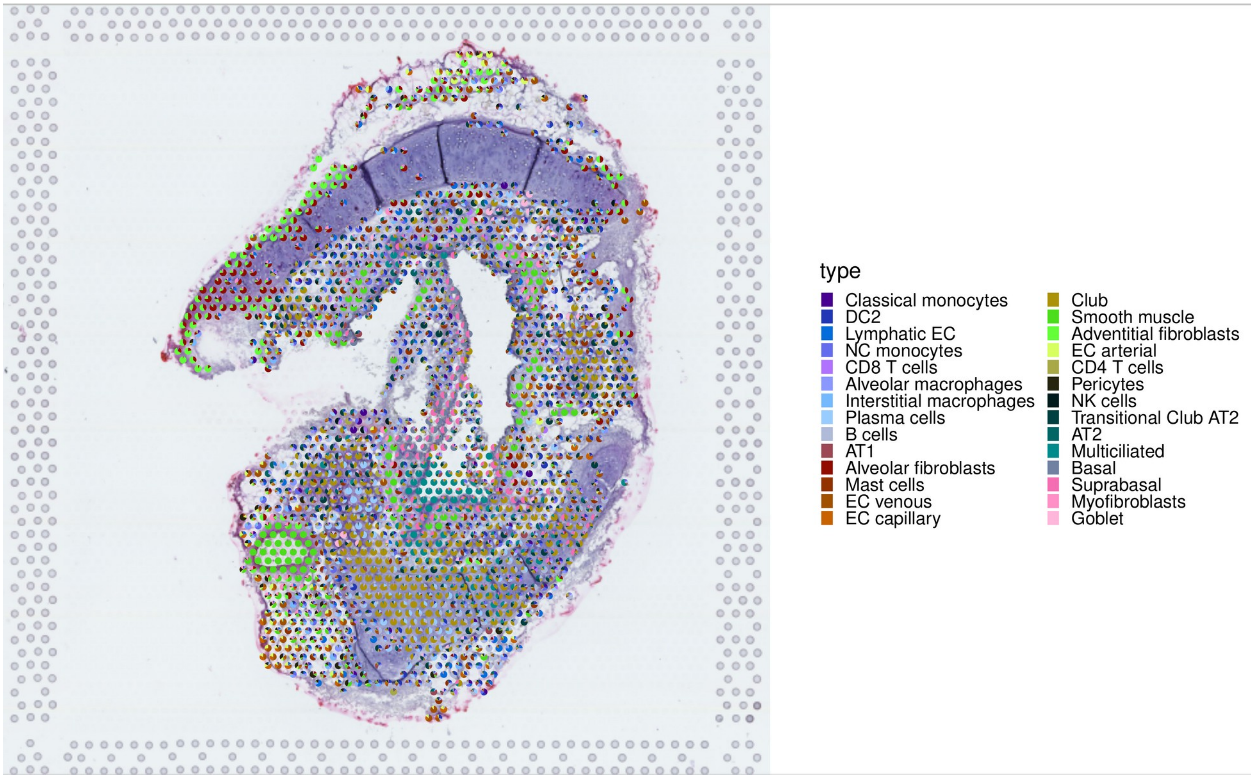

Figure S12. The cell-type composition for never-smoker sample WSA_LngSP8759311 illustrated in a scatterpie plot.

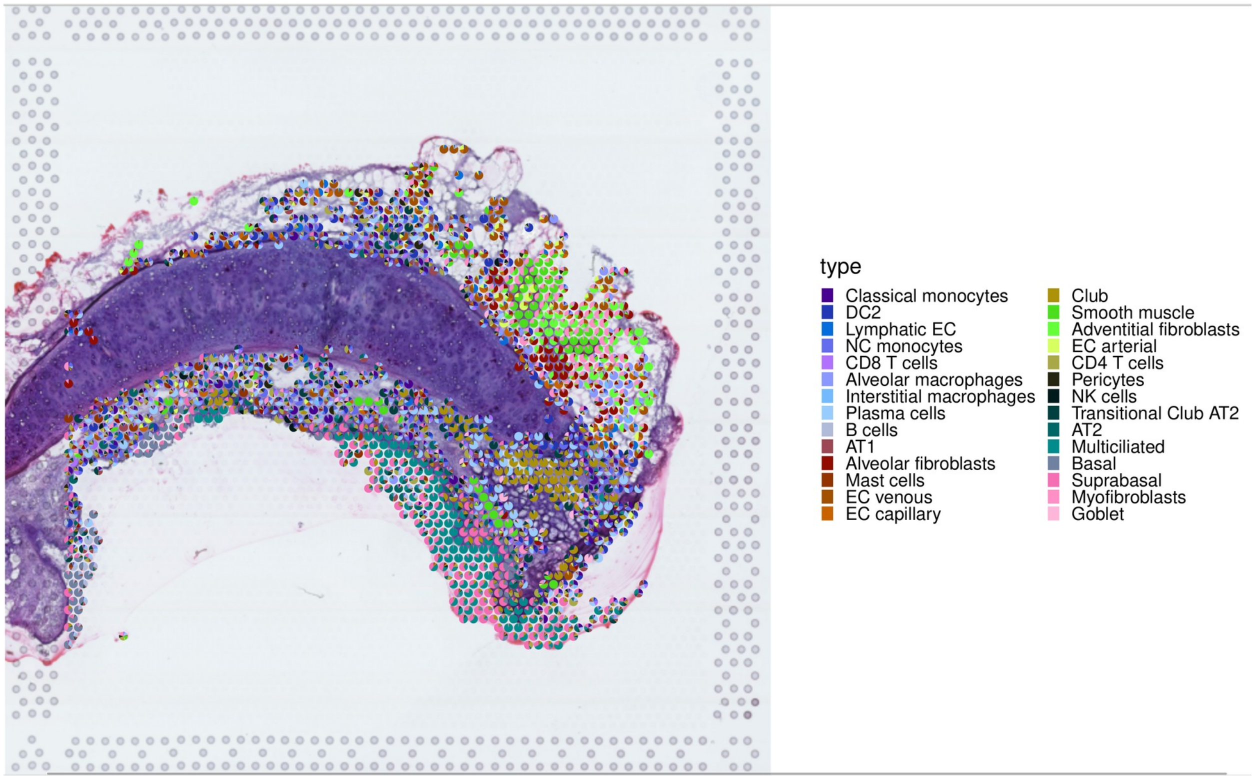

Figure S13. The cell-type composition for never-smoker sample WSA_LngSP8759313 illustrated in a scatterpie plot.

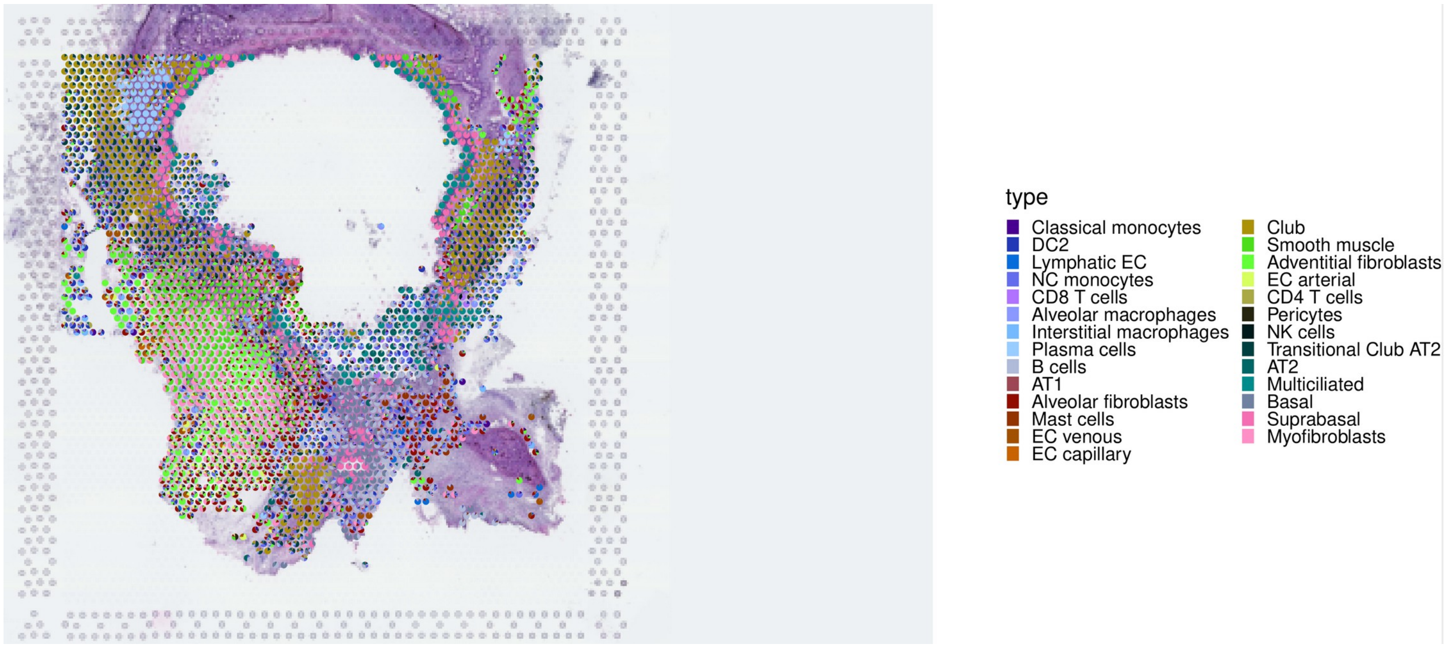

Figure S14. The cell-type composition for current-smoker sample WSA_LngSP9258468 illustrated in a scatterpie plot.

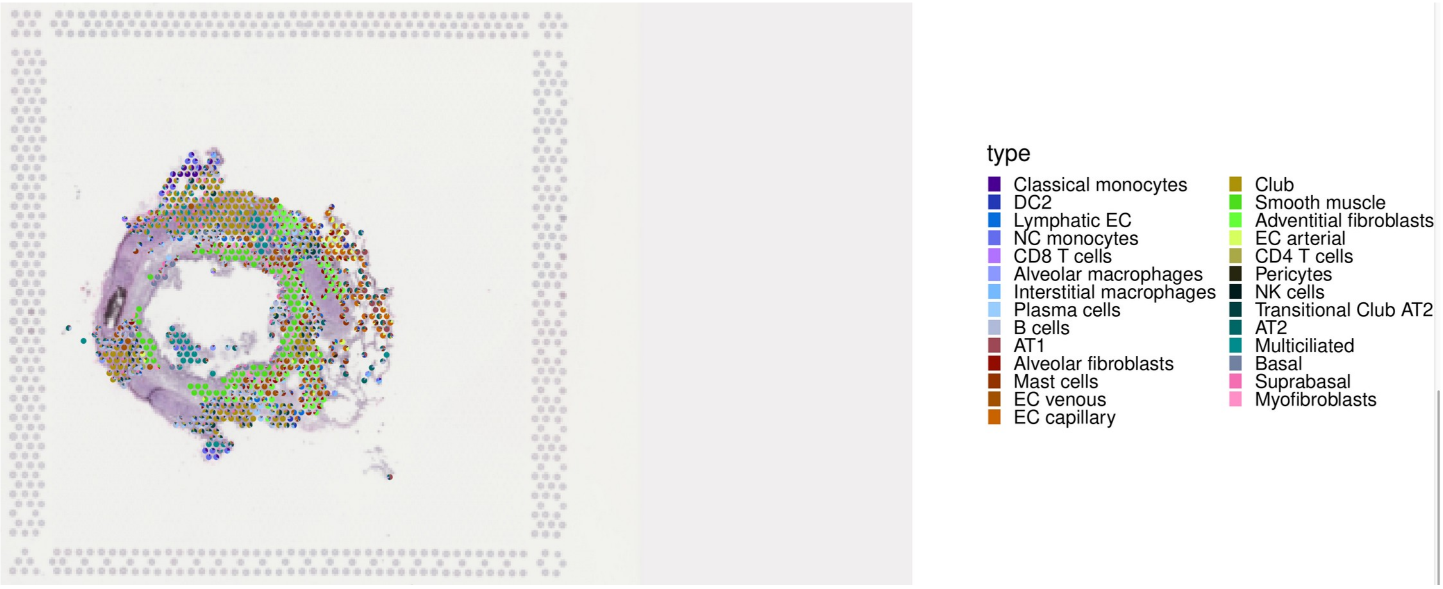

Figure S15. The cell-type composition for current-smoker sample WSA_LngSP10193348 illustrated in a scatterpie plot.

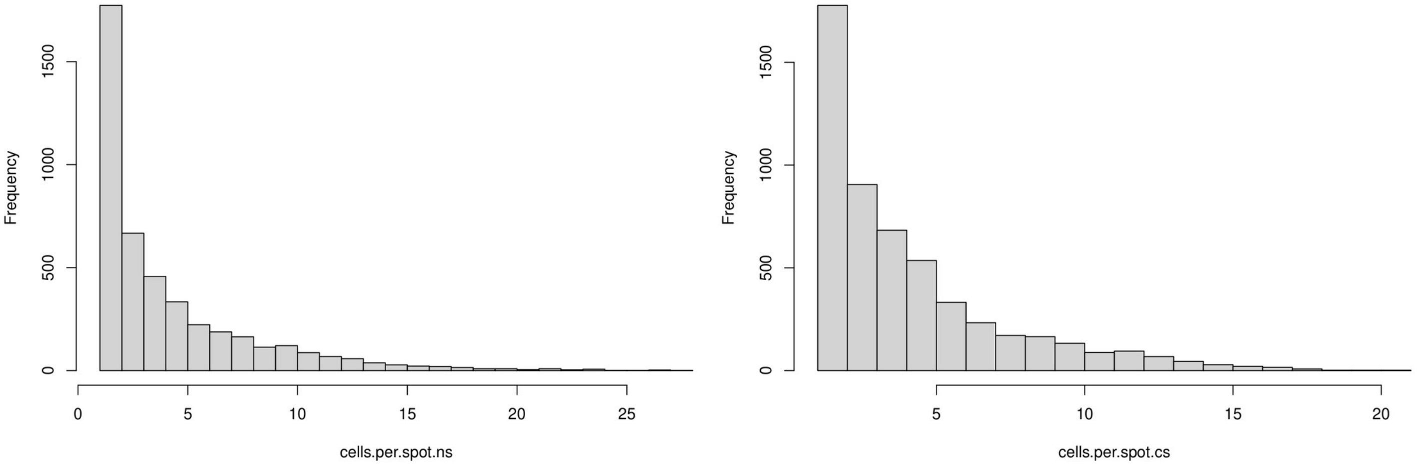

(A) (B)

Figure S16. Histogram of the number of cells mapped to spots in the spatial data for (A) never-smoker samples and (B) current-smoker samples.

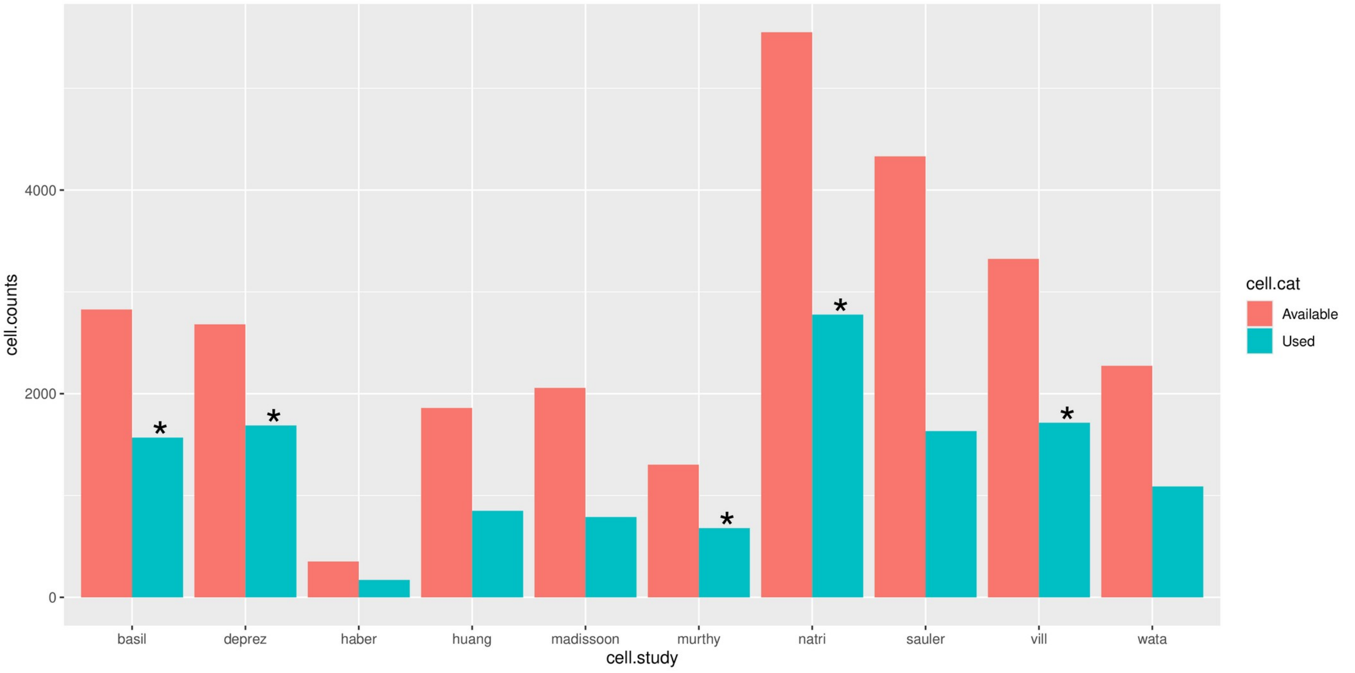

Figure S17. Bar plot of cell usage by CytoSPACE for the never-smoker scRNA-seq atlas data. Significant enrichments ($p < 0.05$) are labeled with an asterisk.

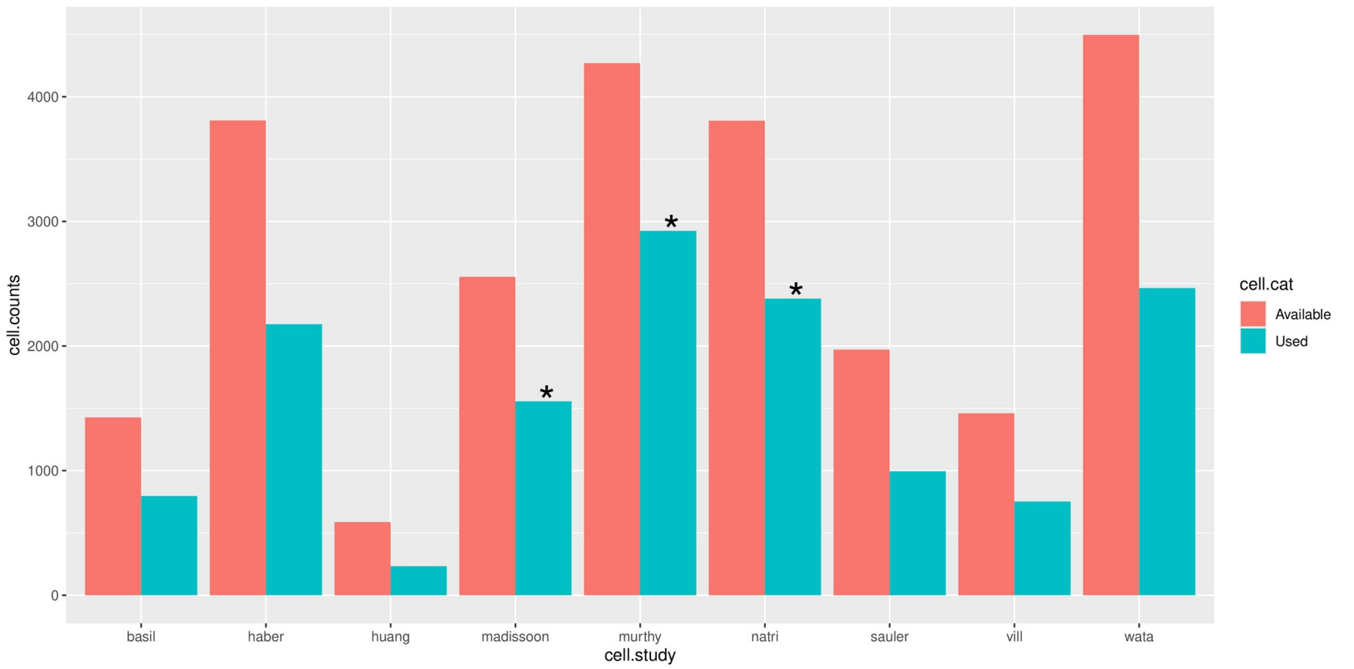

Figure S18. Bar plot of cell usage by CytoSPACE for the ever-smoker scRNA-seq atlas data. Significant enrichments ($p < 0.05$) are labeled with an asterisk.

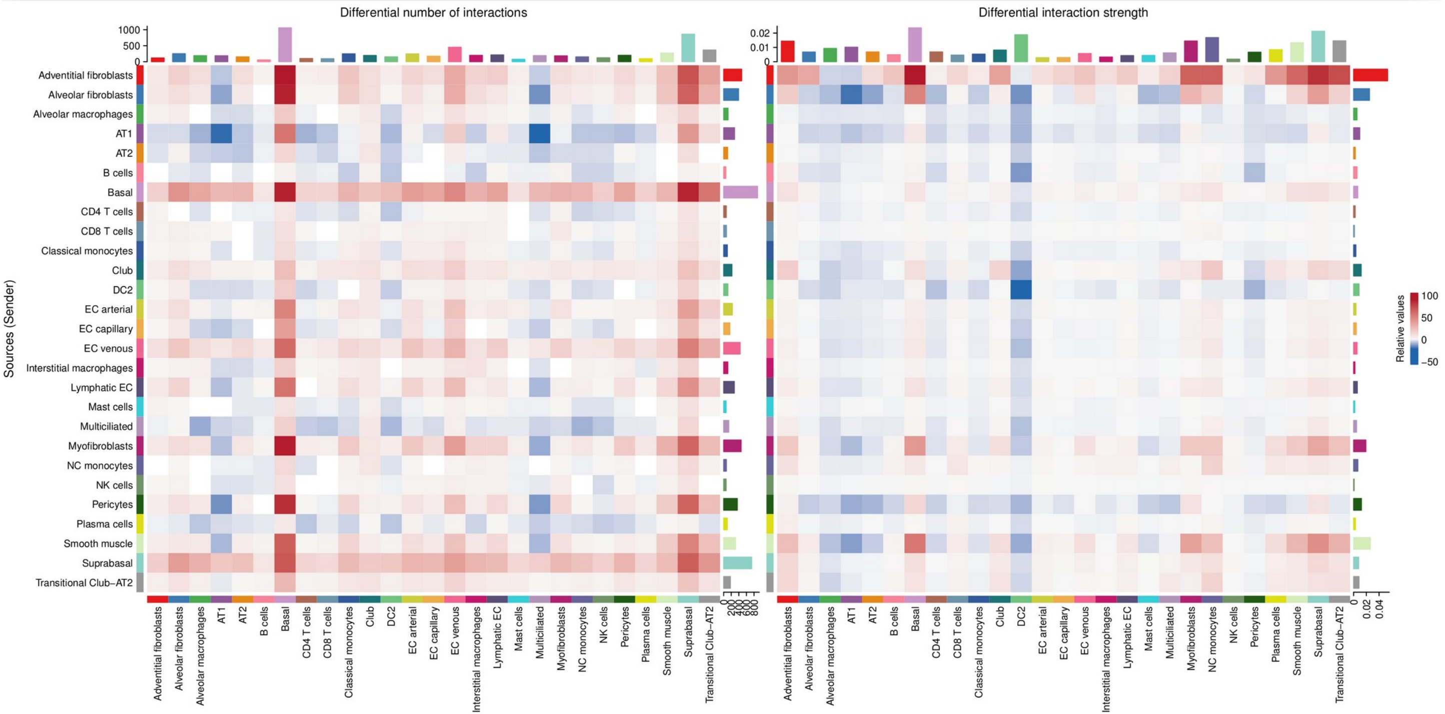

(A) (B)

Figure S19. Heatmaps of the (A) differential number of interactions and (B) the differential interaction strength within the CellChat cell-cell communication network between the two subjects (NS subject vs. CS subject)

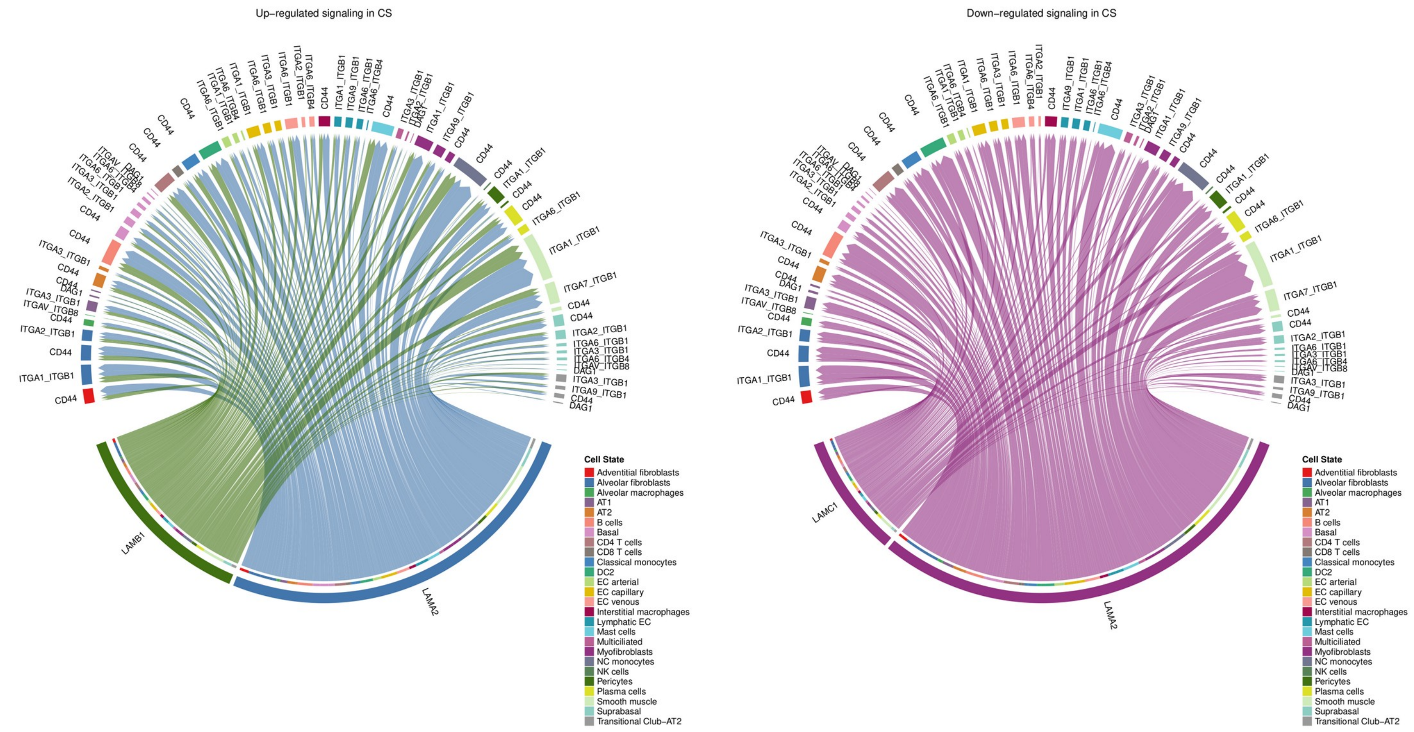

Figure S20. Chord diagrams for (A) the up-regulated signaling pathway LAMININ, and (B) the down-regulated signaling pathway LAMININ.

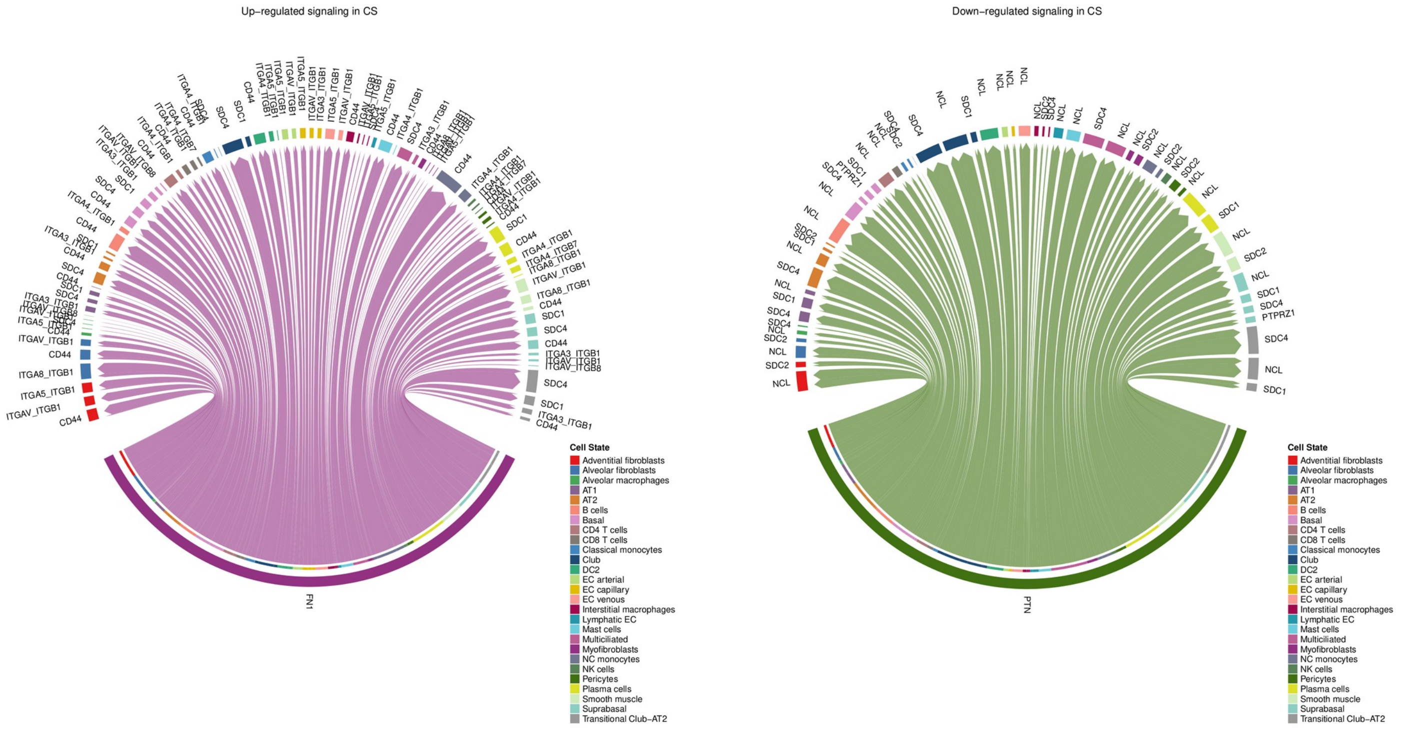

Figure S21. Chord diagrams for (A) the up-regulated signaling pathway FN1 (Fibronectin-1), and (B) the down-regulated signaling pathway PTN (Pleiotrophin).

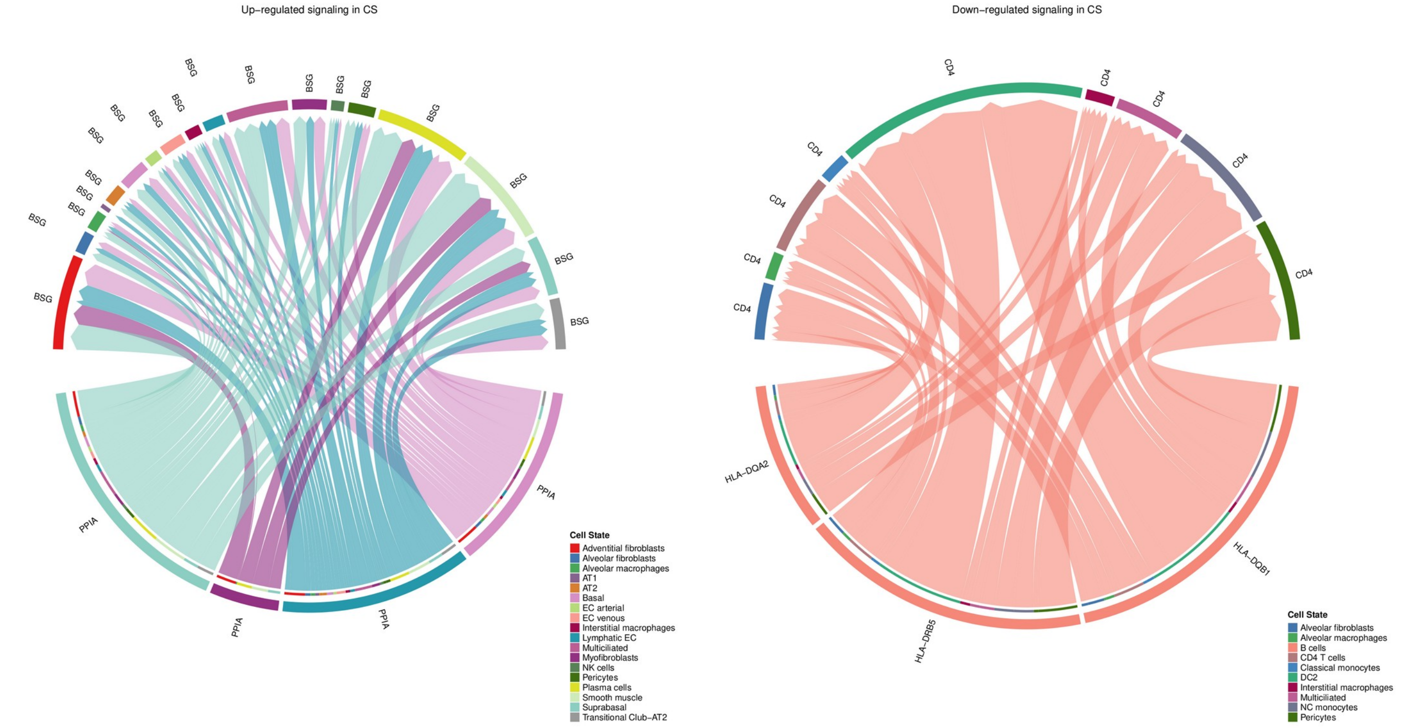

Figure S22. Chord diagrams for (A) the up-regulated signaling pathway CypA (PPIA: Cyclophilin A), and (B) the down-regulated signaling pathway MHC-II.

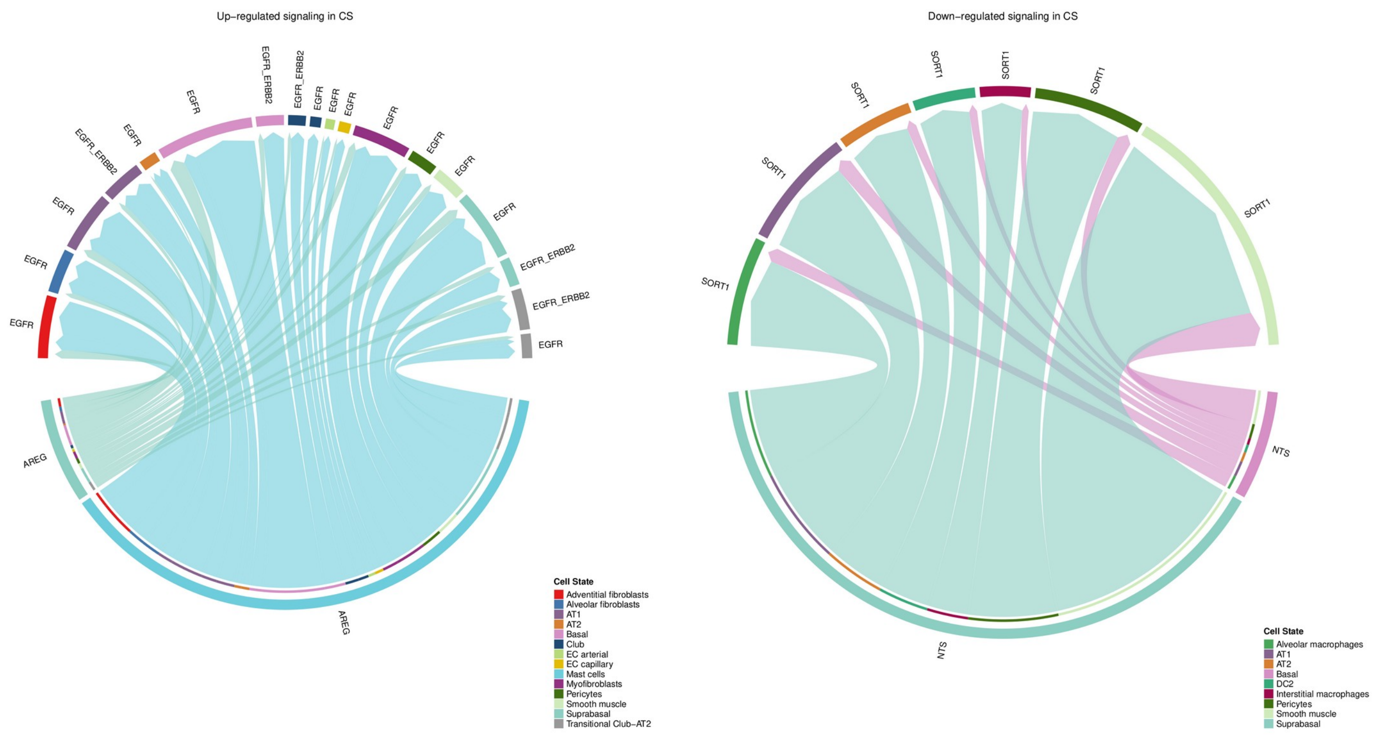

Figure S23. Chord diagrams for (A) the up-regulated signaling pathway EGF (Epidermal growth factor), and (B) the down-regulated signaling pathway NTS (Neurotensin).

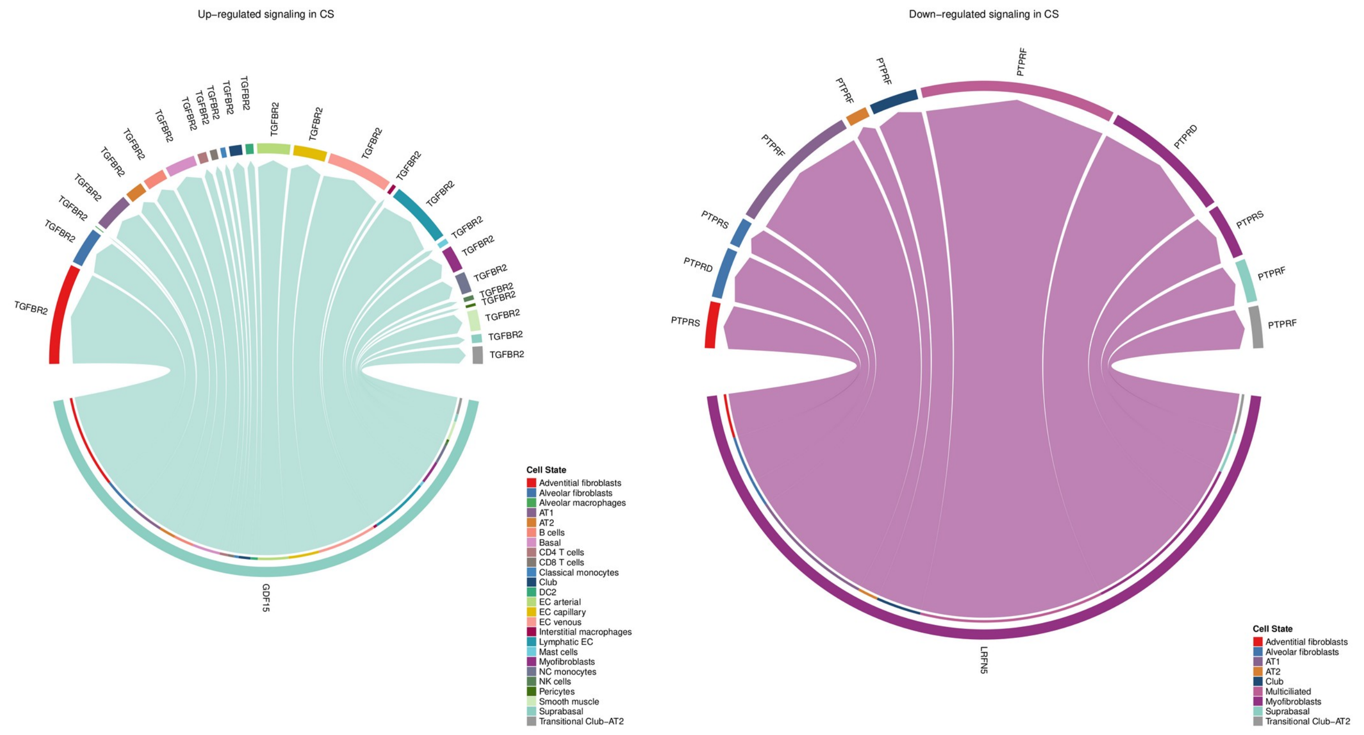

Figure S24. Chord diagrams for (A) the up-regulated signaling pathway GDF (Growth differentiation factor), and (B) the down-regulated signaling pathway PTPR (protein tyrosine phosphatase receptor).

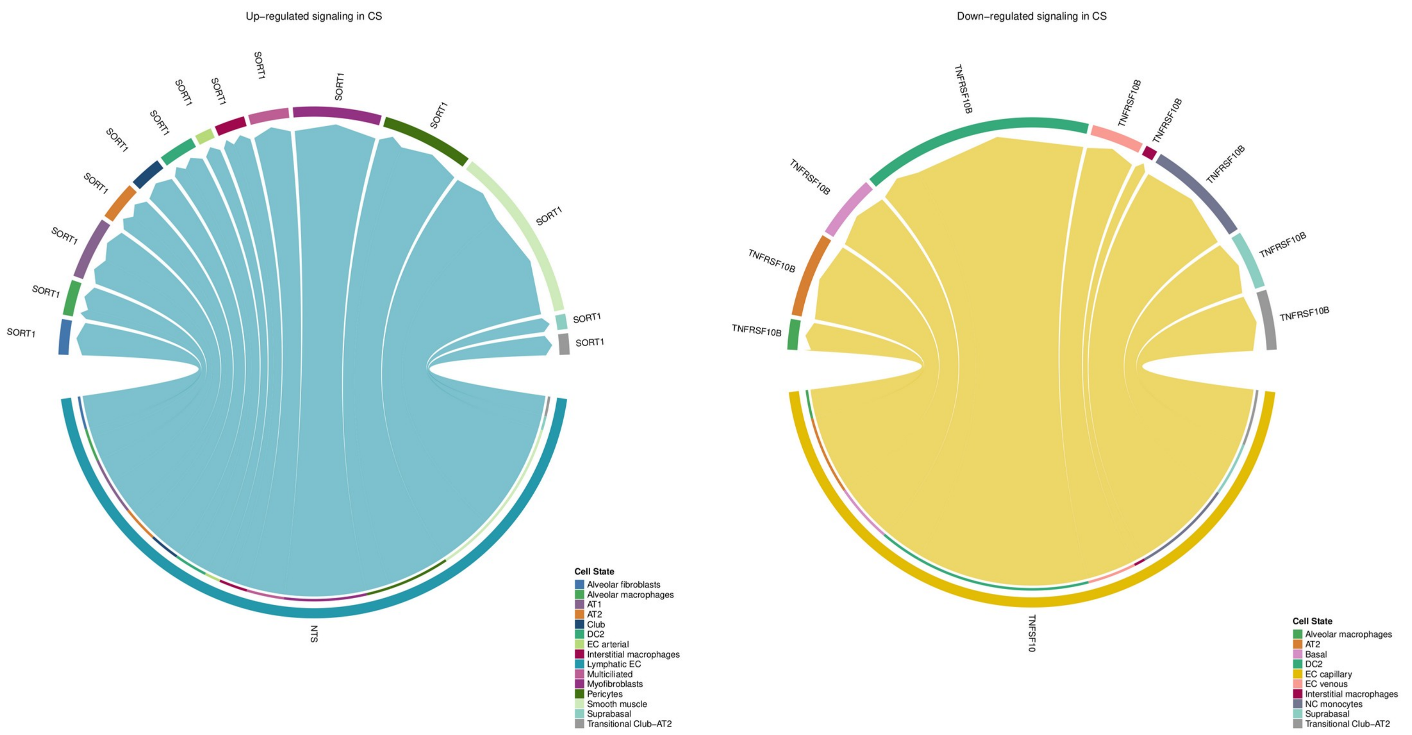

Figure S25. Chord diagrams for (A) the up-regulated signaling pathway NTS (Neurotensin), and (B) the down-regulated signaling pathway TRAIL (tumor necrosis factor-related apoptosis-inducing ligand).

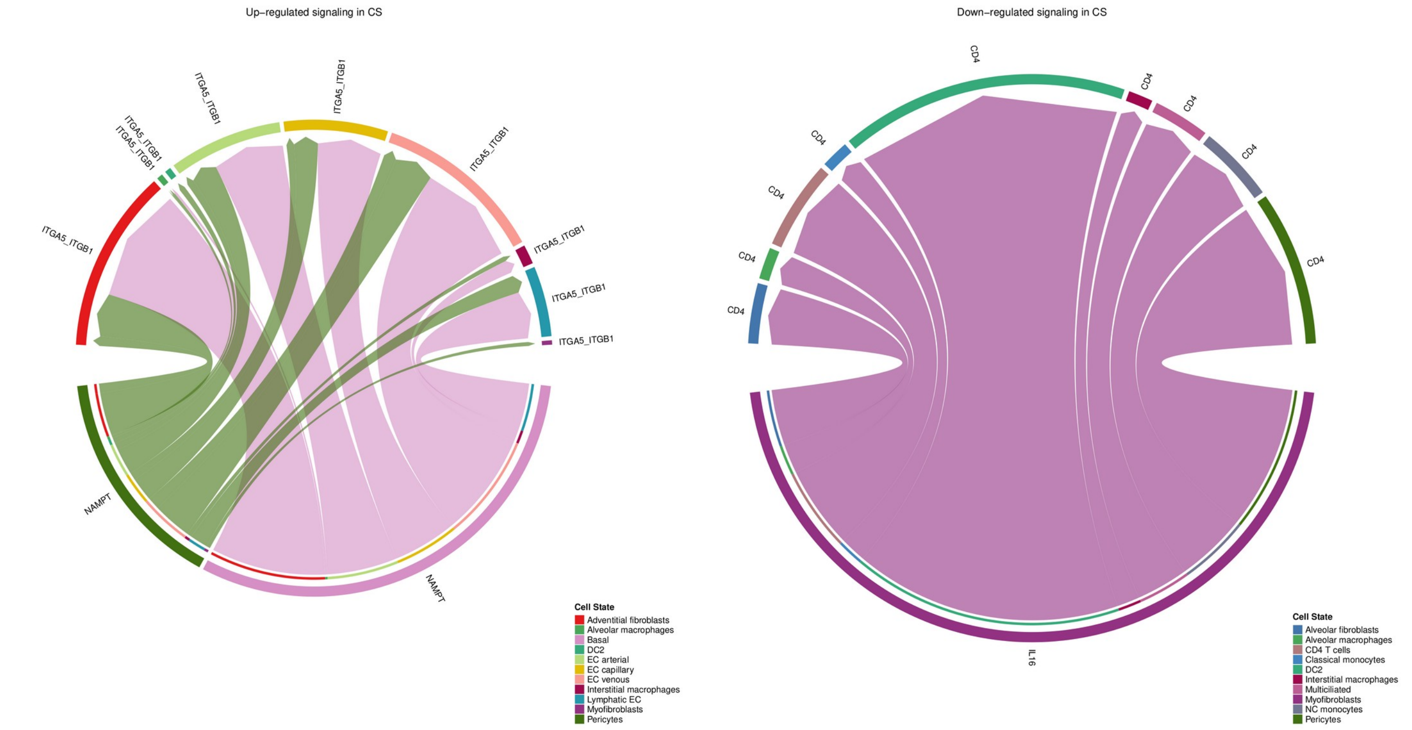

Figure S26. Chord diagrams for (A) the up-regulated signaling pathway VISFATIN, and (B) the down-regulated signaling pathway IL16.
